# Supplementary figures and images for: The use of a chimeric antigen for Plasmodium falciparum and P. vivax seroprevalence estimates from community surveys in Ethiopia and Costa Rica
Source: PLoS One. 2022 May 25;17(5):e0263485. doi: 10.1371/journal.pone.0263485 (PMC9132309; doi:10.1371/journal.pone.0263485)

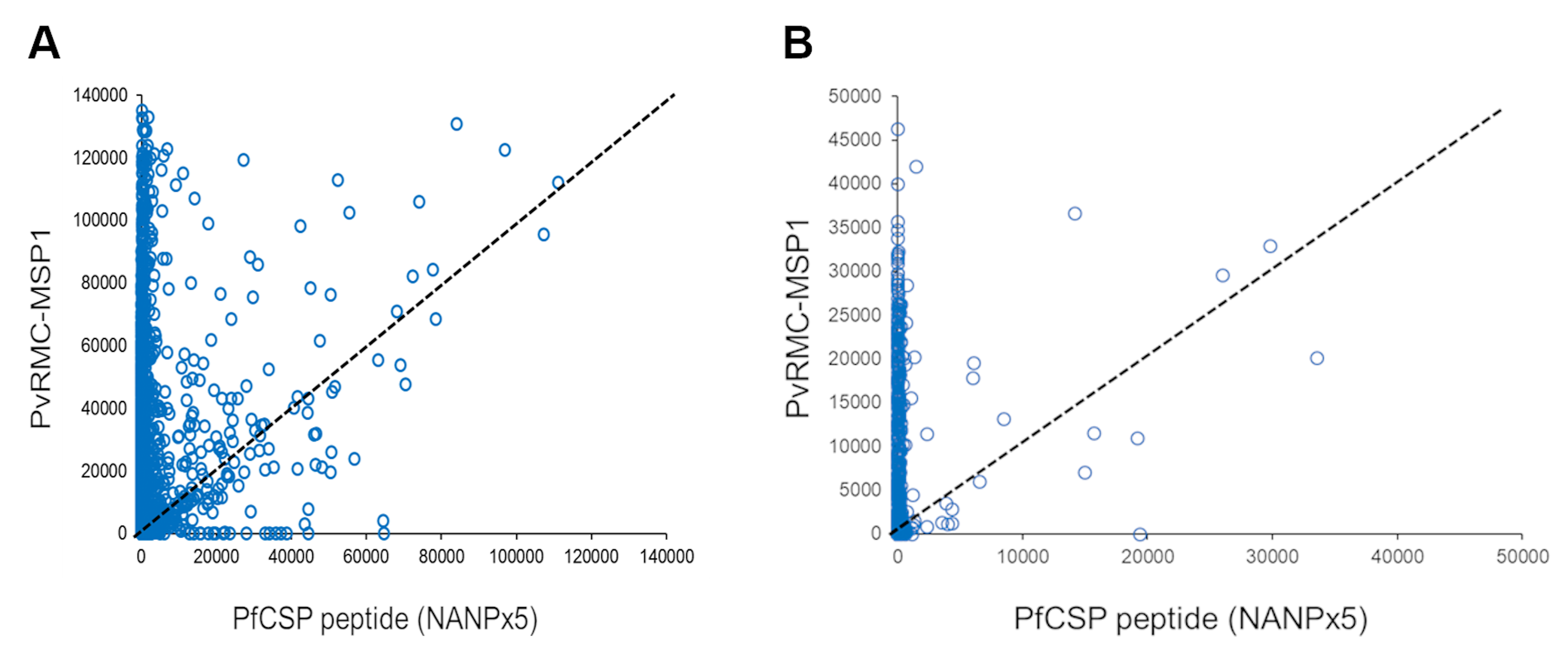

Supplement: S1 Fig — A) Scatterplot of PfCSP signal compared to PvRMC-MSP1 for the Ethiopian study population. B) Scatterplot of PfCSP signal compared to PvRMC-MSP1 for Costa Rican study population. The hashed reference line shown is y = x. (TIF) [file pone.0263485.s001.tif]
