# Supplementary material for: The use of a chimeric antigen for Plasmodium falciparum and P. vivax seroprevalence estimates from community surveys in Ethiopia and Costa Rica
Source: PLoS One. 2022 May 25;17(5):e0263485. doi: 10.1371/journal.pone.0263485 (PMC9132309; doi:10.1371/journal.pone.0263485)
Supplement: Attachment — Submitted filename: McCaffery_etal__PvRMCMSP1_PLOS_One_Response to Reviewers.docx [file pone.0263485.s004.docx]

**Response to Reviewer Comments**

**Manuscript Title:** The use of a chimeric antigen for *Plasmodium falciparum* and *P. vivax* seroprevalence estimates from community surveys in Ethiopia and Costa Rica

**PLOS ONE Submission ID:** PONE-D-22-01720

**Reviewer 1 - Review Comments to the Author:**

1. **Throughout the article the acronym “PvRMC-MSP1” was used a few times for the chimeric malaria antigen however, it would be better to use the term (PvRMC-MSP1/PfCSP) on the whole text, as the use of the first term could lead to a misinterpretation, that the antigen used had only specific targets for vivax.**

We thank the reviewer for this suggestion, but this has previously been the name designated to this antigen by our multiple previous publications, and it should remain the same for consistency. Throughout the manuscript, we state multiple times that PvRMC-MSP1 also contains the epitopes for PfCSP, so this is made clear throughout the whole text for the reader’s interpretation.

2. **In the introduction, it would be better to use the most current reference from the World Health Organization – Malaria Report 2021 (References 1, 17, 18).**

The reviewer is correct that newer references should be used where possible. The World Health Organization Malaria Report citation has been updated to the 2021 version for references 1. The first sentence has also been updated to “In 2021, the World Health Organization reviewed the progress made towards malaria elimination since 2000 and placed increased emphasis on examining the global trends in the burden of malaria to identify any variations in malaria burden by age as malaria transmission declines (1).” to reflect the focus of the most recent WHO Malaria report.

The 2018 Malaria Country Profiles for Ethiopia and Costa Rica are the most recent versions of these reports available for these countries from the WHO.

3. **In the “Materials and Methods - Study sites, design, enrollment, and ethics statements” in relation to Ethiopia, briefly explain how the areas of study were selected. Insert in this topic the total number of participants from Ethiopia.**

The household survey samples obtained from the multiple sites in Ethiopia correspond to the sites included in the 2015 Multiple Indicator Survey. The study was conducted from September 30, to December 10, 2015, covering a sample of 13,875 households in malarious areas between 2,000m and 2,500m above sea level. The goal was to ensure that the survey participants were representative of malaria endemic regions throughout the country. After completion of the main goals of the Multiple Indicator Survey, the remaining 7,077 DBS were made available for serological data collection for this study.

Lines 88-93 have been updated to: “Ethiopian samples were collected as part of the 2015 Ethiopia Malaria Indicator Survey (MIS) (19, 20), a national household survey that occurred between September 30 and December 10, 2015, which coincided with the high malaria transmission season and covered 13,875 households in malarious areas between 2,000m and 2,500m above sea level. Two-stage cluster randomized sampling was used to select 555 areas and 25 households within each area to ensure that the survey participants were representative of malaria endemic regions throughout the country.”

4. **Explain - From the Costa Rica the youngest participant was 12 years old however, in Figure 2b, there are data for children under 12 years old.**

The authors thank the reviewer for suggesting additional clarification in figure 2. In panel A of Figure 2, seropositivity to each antigen is modeled down to age 0 using data collected from Costa Rican participants ages 12 and up. In panel B showing the LOESS regression curves, the x-axis starts at 12 years and the curve stops at 90 years. We have revised the legend for panel B to indicate the x-axis start to avoid confusion: “The x-axis for the LOESS regression curves starts at age 12, corresponding to the youngest participant from the Costa Rican survey.”

5. **In the discussion, in relation to Ethiopia, it would be recommended comparing the data from the different selected areas of the country relating to seroconversion rate and the prevalence of the different species of plasmodium. Use tables and figures to relate this data.**

The authors thank the reviewer for this interesting question. Based on the Ethiopian Public Health Institute’s National Strategic Plan 2021-2025, the most complete and recent aggregated regional data, the *P. falciparum/P. vivax* ratio has maintained at 70/30% for these parasite species for most regions of the country. Even with changes over time, *P. falciparum* has remained the dominant malaria species in Ethiopia. Specifically with serological data, this study examined population exposure over long periods of time due to the longevity of IgG responses. Based on these factors, it would be difficult to correlate temporal differences throughout the country with serological data.
