# Supplementary material for: The use of a chimeric antigen for Plasmodium falciparum and P. vivax seroprevalence estimates from community surveys in Ethiopia and Costa Rica
Source: PLoS One. 2022 May 25;17(5):e0263485. doi: 10.1371/journal.pone.0263485 (PMC9132309; doi:10.1371/journal.pone.0263485)
Supplement: Attachment — Submitted filename: McCaffery_etal_Response to Reviewers_Revision 2.docx [file pone.0263485.s005.docx]

**Response to Reviewer Comments**

**Manuscript Title:** The use of a chimeric antigen for *Plasmodium falciparum* and *P. vivax* seroprevalence estimates from community surveys in Ethiopia and Costa Rica

**PLOS ONE Submission ID:** PONE-D-22-01720

**Review Comments to the Author:**

**Reviewer #1: The authors carefully answered the questions raised above.**

**The modifications contributed to a better understanding of the study.**

**The research described in this article is unprecedented and highly relevant.**

The authors thank the reviewer for their positive feedback of our manuscript.

**Reviewer #2:**

1. **Introduction:**
a. **Line 69: Sorry if I misunderstood, but reference 15 (PMID: 27708348) doesn’t show results about U.S travelers, only 16 (PMID: 33579292).**

The authors apologize for the confusion. Original citation 15 is the original production of the chimeric protein as a vaccine candidate and includes the relevant details regarding design. Original citation 16 is the results of the serology studies from malaria infected US travelers.

We have revised lines 68-72 to include these citations in their appropriate locations.

b. **Additionally, you should mention the results of a seroepidemiological study involving individuals naturally exposed to P. vivax and cross-reactivity in individuals with active infection by P. falciparum from 16 (Figure 6).**

The authors thank the reviewer for this suggestion. Lines 70-74 of the introduction currently state “It was evident from this study that this chimeric antigen captured IgG from a majority of returning U.S. travelers with PCR confirmed malaria infection regardless of the Plasmodium species responsible for infection (15). Furthermore, an increased assay signal was observed for PvRMC-MSP1 compared to recombinant PvMSP1 in 34 out of 38 active P. vivax infections (15).”

c. **Moreover, based on which results did the authors decide to use a P. vivax chimeric protein to evaluate P. falciparum antibodies? It is important to justify. I felt confused reading the introduction.**

Thank you for pointing out that the rationale of this study is not sufficiently clear. Currently the rationale of this study is summarized on lines 65-67: “It remains to be determined if chimeric antigenic constructs from different malaria parasite species can be used to collect serology data for multiple species in serosurveys,” and lines 75-82: “A better understanding of the utility of chimeric antigens for capturing antibody responses in endemic populations with different levels of malaria exposure will help determine future applications of such tools for malaria serological studies. Therefore, this study evaluated the ability of PvRMC-MSP1 to capture IgG from dried blood spot samples (DBS) collected from Ethiopia and Costa Rica. Ethiopia was selected for this study because the country is co-endemic for both P. falciparum and P. vivax, making up 69% and 27% of malaria cases, respectively (17). Costa Rica was selected as a representative low, mono-species Plasmodium endemic region, and as of 2017, 98% of the country was considered malaria-free, with only three remaining foci of active transmission for P. vivax (18).”

It is important to note that we are not suggesting that all anti-*P. falciparum* antibodies are captured by this chimeric antigen since it is based on *P. vivax* MSP1 and the NANP-repeat region of *P. falciparum* CSP. Due to the CSP region of the chimeric protein, as well as the promiscuous B cell epitopes from *P. vivax* MSP1, we have the capacity to capture some amount of anti-*P. falciparum* IgG. We want to emphasize in this publication is that there currently isn’t an ideal antigen that can capture all IgG responses induced following exposure to the different malaria species. To address this gap, we present a proof of concept that an engineered antigen can be used to capture antibody responses from exposure to different malaria species, and that this is a useful approach to consider when developing future engineered antigens. We believe that this idea is sufficiently covered in the discussion.

Regarding the contribution of the *P. vivax* MSP1 portion of the chimera versus the *P. falciparum* CSP NANP repeat region to contribute to the capture of IgG from *P. falciparum*-exposed persons, as part of our previous publication where we investigated the ability of US travelers with active malaria infection to recognize PvRMC-MSP1 as compared to recombinant MSP1 proteins from the four main malaria species, we included in figure 7 a comparison of the PfCSP signal to the PvRMC-MSP1 signal for the 181 individuals with *P. falciparum* infection.

Overall, we found “many samples were double-positive, responding to both of these antigens (Fig. 7). Additionally, some plasma samples showed a correlation of assay signals between the two antigens, tracking on a y = x reference line. However, some of these assay signals from *P. falciparum* infections were non-existent for PfCSP yet showed very high PvRMC-MSP1 IgG binding. No samples were IgG positive to PfCSP alone.”

We interpreted the double positive signals as normal for a population with *P. falciparum* exposure and did not conclude that *PfCSP* was responsible for the signals observed from *P. falciparum* patients as “some of these assay signals from *P. falciparum* infections were non-existent for PfCSP yet showed very high PvRMC-MSP1 IgG binding. No samples were IgG positive to PfCSP alone.”

2. **Material and methods**
a. **86-97: The authors should include the number of individuals enrolled in the study Costa Rica = 7,077 ?**

In lines 165-166 of the results, we state “From the 2015 Ethiopia MIS, 7,077 DBS were available for serological data collection, and 851 DBS were available from the 2015 Costa Rica survey.”.

On line 107 of the methods, we state “In the Costa Rican canton of Matina in 2015, a total of 851 individuals were enrolled in their households and provided blood samples for DBS creation.”

For improved clarity, we have also added this statement to line 106-106: “From the 2015 Ethiopia MIS, 7,077 DBS were available for serological data collection.”

b. **127-134: To clarify. The protocol was performed overnight. The mix reagent containing conjugated antibodies (anti-Human IgG) and samples were added to the well. Did the reaction happen with all components (primary, secondary, and Streptavidin-PE) add at the same time? Why did the authors include IgG4 in to mix reaction?**

The reviewer is correct, the binding of beads to hIgG, hIgG to biotinylated detection Ab, and binding of biotinylated Ab to streptavidin-PE all happens simultaneously when these reagents are mixed together.

The supplementary anti-IgG4-BIOT is added as the anti-hIgG included in the formulation from Southern Biotech does not pick up hIgG4 very well. This was noted from previous experiments and published in 2020 in the AJTMH by Jeffrey W. Priest et al. To explain this better in the manuscript text, we have updated lines 145-147 to include “. Anti-IgG4 was added separately to the anti-human IgG mixture as we have previously found that anti-IgG4 responses are difficult to determine using only the standard anti-IgG cocktail,” and cited this publication.

c. **138: MFI signal was generated for a target of 50 beads/region = 50 beads per antigen?**

The reviewer is correct that a minimum of 50 beads per region (fluorescent signal) are required by the Luminex MagPix to produce signal, as stated in line 149 “MFI signal was generated for a target of 50 beads/region.”. Region in this case is the pre-gated bead region, or fluorescence signal from each bead type used in the assay. We separately conjugate each fluorescently tagged bead obtained from the manufacturer to one antigen of interest to allow us to obtain unique signals for PvRMC-MSP1 vs recombinant PvMSP1 vs recombinant PfMSP1. The multiplex platform allows us to obtain the level of antibody recognizing each antibody simultaneously in one well.

3. **Results**
a. **153-156: The authors should describe data from Table1 according to table data. First, Costa Rica and, than Ethiopia.**

As suggested, we have revised table 1 to include the demographic data from Ethiopia first, above that of Costa Rica, to keep discussion of Ethiopia and then Costa Rica consistent throughout the manuscript.

b. **162-164: I suggest the authors a table with seroprevalence data according to the region to make easy the reading of data.**

We thank the reviewer for this interesting question. It is possible to plot and compare the seroprevalence of antibodies to each of the three MSP1 antigens tested by region within Ethiopia, but we believe that this is outside of the scope of this study. Instead, we want to emphasize the seroprevalence to each antigen in the population as a whole, especially since Costa Rica is approaching elimination and our samples were obtained from only one region of Costa Rica.

c. **194-205: NANP6 or NANPx5 peptide?**

The reviewer is correct that this passage is somewhat confusing. We have revised accordingly.

The PvRMC-MSP1 protein contains six copies of the NANP repeat peptide from the *P. falciparum* circumsporozoite protein, abbreviated as (NANP)_6_ on lines 124-125: “the (NANP)_6_ repeat peptide from P. falciparum circumsporozoite protein (CSP)”. This is also indicated on lines 211-214 “Although PvRMC-MSP1 is based primarily on the P. vivax Belem sequence, six copies of the NANP repeat sequence from the P. falciparum circumsporozoite protein (PfCSP) are present at the C-terminus of PvRMC-MSP1 and were included during the design of PvRMC-MSP1 to serve as an additional purification tag.”

Additionally, we have revised lines 214-216 to state “To determine if antibody recognition and binding to the PfCSP region is responsible in part for the assay signals observed for PvRMC-MSP1, the MFI-bg signals were compared between the chimera and a peptide containing five copies of the PfCSP NANP peptide (NANPx5, S1 Figure).”

4. **Discussion**
   a. **241-247: To clarify, the NANP sequence was used as a tag to improve the purification o protein (NANP-6, was included at the C-terminus for biochemical characterization of antigenic integrity and to provide an optimal affinity purification tag, ref 15).**

**In previously published works (refs 15 and 16), a cross-reactivity with P. falciparum was described. However, it was demonstrated that this reactivity is not due to the presence of the PfCSP NANP repeat region alone. How do the authors justify the importance of this sequence on the reactivity of antibodies in this work? I felt confused When I read previous data.**

Thank you for taking the time to look into our relevant previous publications as part of your review of this current manuscript.

The reviewer is correct that the NANP sequence was initially included in the PvRMC-MSP1 protein as a purification tag during its design as a vaccine candidate. In the publication where we investigated the ability of US travelers with active malaria infection to recognize PvRMC-MSP1 as compared to recombinant MSP1 proteins from the four main malaria species, we included in figure 7 a comparison of the PfCSP signal to the PvRMC-MSP1 signal for the 181 individuals with *P. falciparum* infection.

Overall, we found “many samples were double-positive, responding to both of these antigens (Fig. 7). Additionally, some plasma samples showed a correlation of assay signals between the two antigens, tracking on a y = x reference line. However, some of these assay signals from *P. falciparum* infections were non-existent for PfCSP yet showed very high PvRMC-MSP1 IgG binding. No samples were IgG positive alone to PfCSP.”

We interpreted the double positive signals as normal for a population with *P. falciparum* exposure and did not conclude that *PfCSP* was responsible for the signals observed from *P. falciparum* patients as “some of these assay signals from *P. falciparum* infections were non-existent for PfCSP yet showed very high PvRMC-MSP1 IgG binding. No samples were IgG positive alone to PfCSP.”
